# Marginal role of von Willebrand factor-binding protein and coagulase in the initiation of endocarditis in rats with catheter-induced aortic vegetations

**DOI:** 10.1080/21505594.2018.1528845

**Published:** 2018-10-13

**Authors:** Stefano Mancini, Frank Oechslin, Carmen Menzi, Yok Ai Que, Jorien Claes, Ruth Heying, Tiago Rafael Veloso, Thomas Vanassche, Dominique Missiakas, Olaf Schneewind, Philippe Moreillon, José Manuel Entenza

**Affiliations:** a Department of Fundamental Microbiology, University of Lausanne, Lausanne, Switzerland; b Department of Intensive Care Medicine, Bern University Hospital, Bern, Switzerland; c Cardiovascular Developmental Biology, Department of Cardiovascular Sciences, KU Leuven, Leuven, Belgium; d Center for Molecular and Vascular Biology, Department of Cardiovascular Sciences, KU Leuven, Leuven, Belgium; e Department of Microbiology, University of Chicago, Chicago, IL, USA

**Keywords:** Clumping factor A, endocarditis, staphylocoagulase, *Staphylococcus aureus*, von Willebrand factor-binding protein

## Abstract

*Staphylococcus aureus* is the leading cause of infective endocarditis (IE). While the role of *S. aureus* cell-wall associated protein clumping factor A (ClfA) in promoting IE has been already demonstrated, that of the secreted plasma-clotting factors staphylocoagulase (Coa) and von Willebrand factor-binding protein (vWbp) has not yet been elucidated. We investigated the role of Coa and vWbp in IE initiation in rats with catheter-induced aortic vegetations, using *Lactococcus lactis* expressing *coa, vWbp, clfA* or *vWbp/clfA*, and *S. aureus* Newman Δ*coa*, Δ*vWbp*, Δ*clfA or* Δ*coa/*Δ*vWbp/*Δ*clfA* mutants. *vWbp*-expression increased *L. lactis* valve infection compared to parent and *coa*-expressing strains (incidence: 62%, versus 0% and 13%, respectively; *P *< 0.01). Likewise, expression of *clfA* increased *L. lactis* infectivity (incidence: 80%), which was not further affected by co-expression of *vWbp*. In symmetry, deletion of the *coa* or *vWbp* genes in *S. aureus* did not decrease infectivity (incidence: 68 and 64%, respectively) whereas deletion of *clfA* did decrease valve infection (incidence: 45%; *P *= 0.03 versus parent), which was not further affected by the triple deletion Δ*coa/*Δ*vWbp/*Δ*clfA* (incidence: 36%; *P *> 0.05 versus Δ*clfA* mutant). Coa does not support the initial colonization of IE (in *L. lactis*) without other key virulence factors and vWbp contributes to initiation of IE (in *L. lactis*) but is marginal in the present of ClfA.

## Introduction

*Staphylococcus aureus* is the most common etiological agent of infective endocarditis (IE) owing to its propensity to adhere to fibrin-platelet clots on damaged cardiac valves, known as vegetations [1,2]. Several structures in the *S. aureus* envelope, including teichoic acids and surface-associated proteins, have been implicated in ligand-receptor interactions of circulating bacteria to cardiac vegetations and contributed to the pathogenesis of IE [3–5].

The study of gene inactivation in *S. aureus* has greatly contributed to elucidate the specific role of individual virulence factors in the pathogenesis of IE [6]. This has been more recently complemented by the use of the poorly pathogenic *Lactococcus lactis*, used as a surrogate carrier, to investigate the effects of heterologous gene expression on infectivity [7]. Using these methods cooperatively, it was shown that fibrinogen-binding protein clumping factor A (ClfA) and fibronectin-binding protein A (FnBPA, which also binds fibrinogen) were major determinants of *S. aureus* valve colonization [4,6,8–10]. For instance, while *S. aureus* mutants defective in *clfA* became less pathogenic, non-virulent *L. lactis* expressing recombinant *clfA* demonstrated increased infectivity in rats with experimental IE, due, amongst others, to the acquisition of the ability to adhere to fibrin deposited on damaged valve tissues [6,7].

In addition to cell-wall anchored adhesins, *S. aureus* also produces two procoagulant factors, namely staphylocoagulase (Coa) [11] and von Willebrand factor-binding protein (vWbp) [12], that can activate prothrombin in a non-proteolytic manner [13]. These proteins trigger the polymerization of fibrinogen into fibrin and promote platelet aggregation and blood clotting [13–15].

Contemporary studies showed that Coa and vWbp might contribute to the pathogenesis of *S. aureus* in murine and rabbit models of abscess formation, blood stream infection and endovascular colonization [16–19]. Nevertheless, their role in the pathogenesis of *S. aureus* IE remains unclear. On the one hand, two pioneer studies performed in rats with catheter-induced aortic vegetations reported that Coa was not involved in the early colonization of damaged valves [6,20] but, since at the time the presence of vWbp was still unknown [12], the lack of Coa activity in *coa*-defective mutants might have been compensated for by that of vWbp. Moreover, one study using heterologous expression of *coa* in *Streptococcus gordonii* suggested that Coa did not contribute to infectivity, further supporting the notion that Coa was not implicated in the initiation of IE [21].

On the other hand, taking the two procoagulant factors into account, Panizzi and co-workers demonstrated that knocking out both of them in *S. aureus* significantly increased mice survival in a model of *S. aureus* IE [22]. However, this study did not investigate the ability of the bacteria to initiate IE.

The aim of the present work was to provide new insights into the specific roles of Coa and vWbp in the initiation of experimental IE due to *S. aureus* in the rat model of catheter-induced aortic vegetations. To this end we used a series of knock-in and knock-out mutants including (i) *L. lactis* heterologously expressing *S. aureus coa, vWbp, coa* plus *vWbp, clfA*, and *vWbp* plus *clfA*, and (ii) *S. aureus* Newman mutants inactivated in the *coa, vWbp, clfA*, and *coa/vWbp/clfA* genes.

## Results

### Recombinant L. lactis strains produce functional Coa and vWbp

In order to verify that the recombinant *L. lactis* strains produced functional procoagulant *S. aureus* Coa and vWbp proteins, we assessed both their abilities to induce blood clotting and to trigger platelet aggregation.

**Figure 1.**
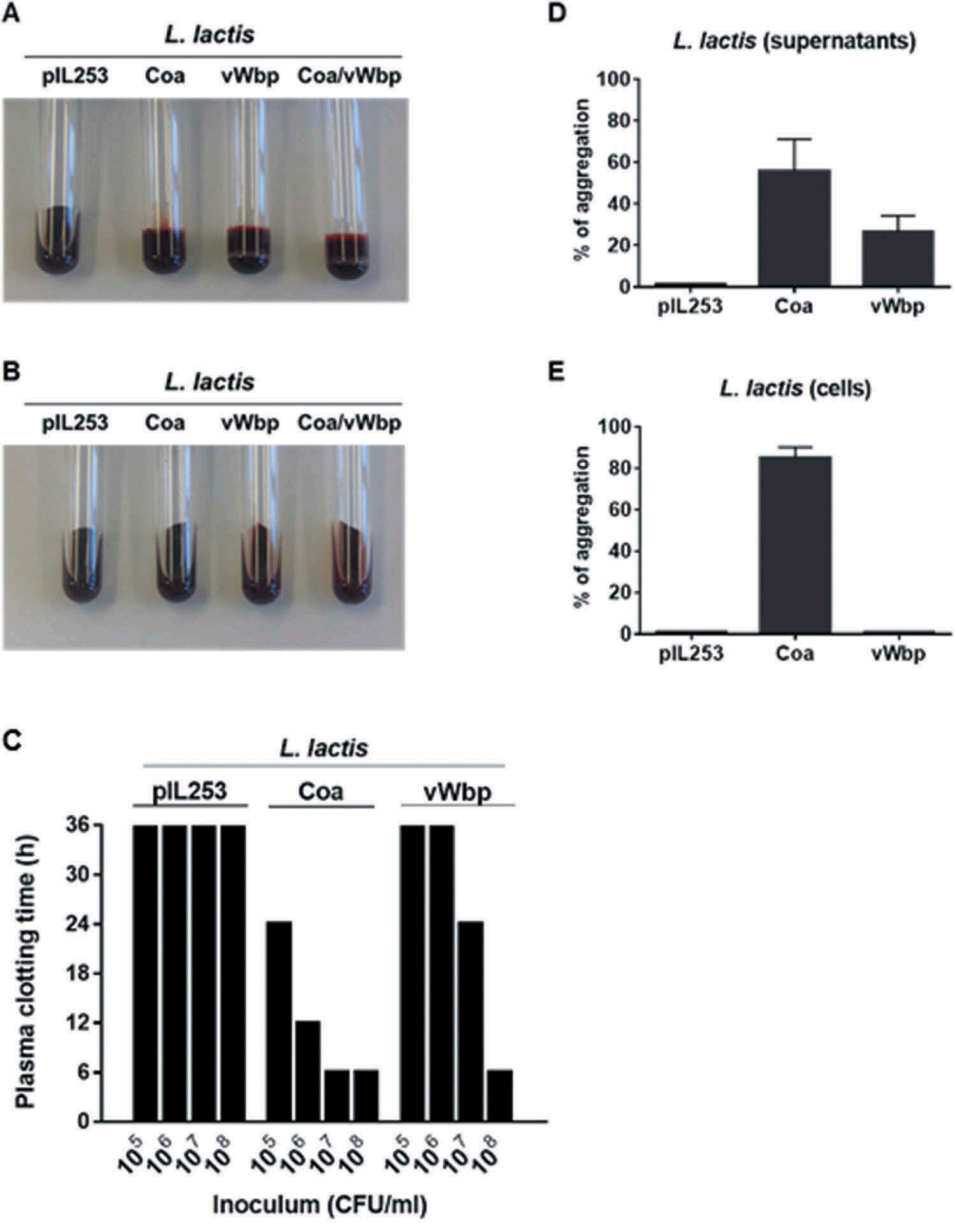
Functional analysis of recombinant *L. lactis* Coa and *L. lactis* vWbp. The activity of Coa and vWbp was evaluated by blood clotting, time required to induce plasma clotting and platelet aggregation. For blood clotting, citrated rat blood without (a) and with (b) dabigatran was infected with 10^7^ CFU of either parent *L. lactis* pIL253, *L. lactis* Coa, *L. lactis* vWbp or *L. lactis* Coa/vWbp cells from an overnight culture and incubated 24 h at 37°C. Tubes were tilted to display blood coagulation. Data are representative of three independent experiments. To assess the time required to induce plasma clotting (c), 10^5^ to 10^8^ CFU of parent *L. lactis* pIL253, recombinant *L. lactis* Coa or *L. lactis* vWbp were incubated at 37°C with rat plasma. Tubes were tilted to assess plasma coagulation at 4, 8, 12, 24 and 36 h. Clotting values of 36 h, the maximal time of measurement, indicate that there was no clotting. Data are representative of three independent experiments. For platelet aggregation, cell-free supernatants (d) or pelleted cells (e) of parent *L. lactis* pIL253, or recombinant *L. lactis* Coa or *L. lactis* vWbp from overnight cultures were mixed with platelet rich plasma. Platelet aggregation was monitored by aggregometry and the maximum values of light transmission (maximal aggregation) reached over 20 min incubation were recorded. Data are expressed as mean ± standard deviation (SD) of three independent assays.

As shown in Figure 1(a), all *L. lactis* strains expressing the *S. aureus* pro-coagulant factors, but not the parent *L. lactis* pIL253 strain, exhibited blood clotting activity. Consistently, dabigatran prevented blood clot formation by recombinant *L. lactis* strains (Figure 1(b)). Moreover, it took 100-times less lactococci producing Coa than vWbp to trigger coagulation within 24 h (Figure 1(c)).

Since both *S. aureus* Coa and vWbp are able to induce platelet aggregation [13,23], the capacity of Coa and vWbp *L. lactis* recombinants to trigger platelet aggregation was also tested. Moreover, since a previous study by McDevitt and collaborators showed that a fraction of Coa was retained on the surface of *S. aureus* [24], the ability of the *L. lactis* Coa and *L. lactis* vWbp cell-free supernatants and cells to induce platelet aggregation was also tested to verify whether Coa and vWbp were also partially associated to the surface of lactococci. As shown in Figure 1(d), platelet aggregation was observed upon addition of the cell-free supernatants of *L. lactis* expressing *coa* and *vWbp*, but not upon the addition of the supernatants of the parent *L. lactis* pIL253. When cells were used, only Coa-positive lactococcal cells exhibited platelet aggregation activity (Figure 1(e)).

Collectively, these results indicate that the recombinant *L. lactis* strains produced and secreted functional *S. aureus* Coa and vWbp and that part of Coa, but not vWbp, was attached to the surface of *L. lactis*.

### Expression of vWbp but not Coa supports L. lactis infection of aortic vegetations in rats

**Figure 2.**
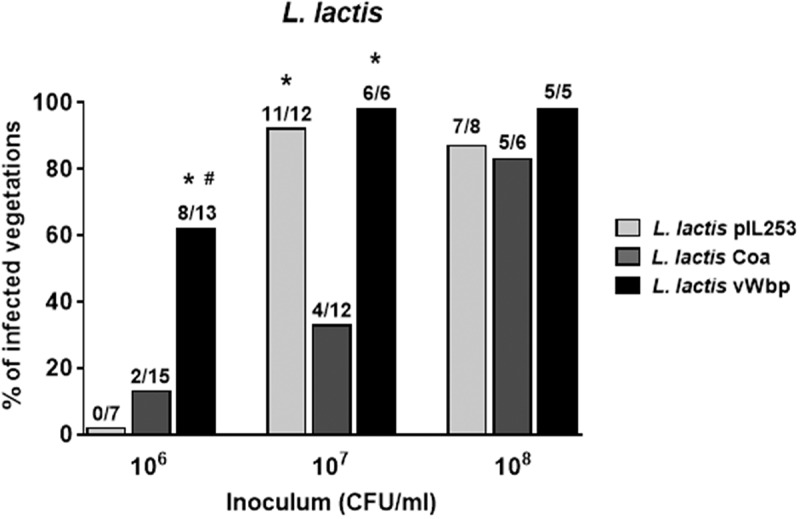
*vWbp* but not *coa* confers *L. lactis* the ability to infect pre-existing sterile valve vegetations. Infectivity titration of the *L. lactis* test organisms in the rat model of experimental endocarditis. The rats were challenged with bacterial inocula of gradually increasing sizes. The columns indicate the percentage of positive vegetations 24 h after bacterial challenge. The number of infected/total number of animals per group is indicated at the top of the columns. *, *P *< 0.05 compared with *L. lactis* Coa; #, *P *< 0.05 compared with *L. lactis* pIL253, as determined by the Fisher’s exact test with the Yates correction.

To study the effect of *coa* and *vWbp* expression on lactococcal infectivity, we titrated the ability of parent *L. lactis* pIL253 and recombinant *L. lactis* Coa, *L. lactis* vWbp and *L. lactis* Coa/vWbp to induce experimental IE in rats. As shown in Figure 2, 24 h after challenge, in animals inoculated with 10^6^ CFU, the percentage of vegetations infected with *L. lactis* vWbp was significantly higher (62%) than that infected with the parent *L. lactis* pIL253 (0%; *P *< 0.01) and *L. lactis* Coa (13%; *P *= 0.007). Not indicated in the Figure, the percentage of vegetations infected with *L. lactis* expressing both *coa* and *vWbp* was similar (62%) to that of *L. lactis* vWbp.

When animals were inoculated with 10-times more bacterial cells (10^7^ CFU), *L. lactis* Coa remained surprisingly poorly infective (33% of endocarditis), while *L. lactis* pIL253 and *L. lactis* vWbp were able to infect ≥ 90 % of vegetations.

Of note, the mean bacterial densities ± standard deviation (SD) in the animals that developed endocarditis (3.58 ± 0.71 log_10_ CFU/g) in animals challenged with *L. lactis* Coa were significantly lower (*P *< 0.001; one-way ANOVA) from that of *L. lactis* pIL253 (5.45 ± 0.78 log_10_ CFU/g) and *L. lactis* vWbp (7.70 ± 0.40 log_10_ CFU/g). At higher bacterial inocula (10^8^ CFU) all *L. lactis* strains were able to infect ≥ 80% of vegetations. In animals that developed endocarditis, the mean bacterial densities in the vegetations with *L. lactis* Coa (5.92 ± 0.67 log_10_ CFU/g) were similar to that with *L. lactis* pIL253 (5.46 ± 0.82 log_10_ CFU/g) but significantly lower (*P *< 0.01; one-way ANOVA) to that with *L. lactis* vWbp (7.75 ± 0.44 log_10_ CFU/g).

In sum, these results showed that vWbp but not Coa increased the infectivity of *L. lactis*, supporting a role of vWbp in the initiation of IE. On the contrary, Coa production paradoxically decreased the ability of lactococci to colonize aortic vegetations.

### Coa promotes the clearance of L. lactis from the vegetations

**Figure 3.**
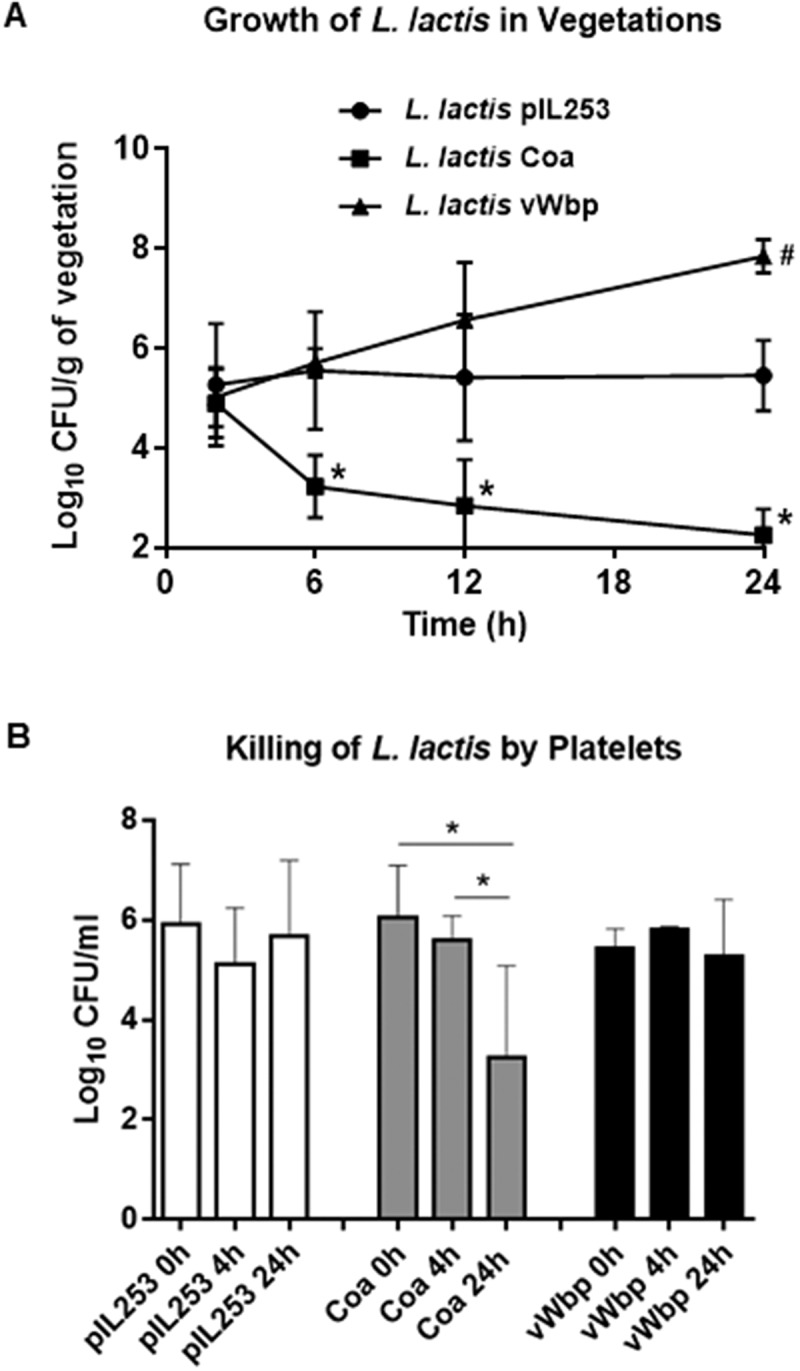
Evolution of vegetation infection after challenge with parent *L. lactis* pIL253 and recombinant *L. lactis* Coa or *L. lactis* vWbp (a) and in vitro killing by platelets (b). (a) Groups of 6–10 rats were inoculated with of 10^7^ CFU and sacrificed at various times after challenge. Data represent mean ± SD of vegetation bacterial titres. *, *P *< 0.01 vs *L. lactis* pIL253 and *L. lactis* vWbp; ^#^ *P *< 0.01 vs *L. lactis* pIL253, as compared by the one-way ANOVA followed by Tukey’s multiple comparisons test. (b) Killing of *L. lactis* by platelets after 4 and 24 h of exposure. *, *P *< 0.05 by one-way ANOVA followed by Tukey’s multiple comparisons test.

Previous work by Bayer et al. [25] showed that the hyperproduction of alpha-toxin by *S. aureus* resulted in a paradoxically reduced virulence in experimental endocarditis. Moreover, they demonstrated that the reduced *in vivo* virulence of the alpha-toxin-producer variant was not due to differences in initial adherence to sterile vegetations compared to the parental strain, and suggested a role of platelet-induced bacterial killing in the vegetations [25]. We investigated whether the same scenario could apply to the lower infectivity rate observed with *L. lactis* Coa in animals challenged with the 10^7^ CFU inoculum. Thus, in a second set of experiments we followed disease progression by infecting rats with 10^7^ CFU of each lactococcal recombinant, and assessed the evolution of valve infection in groups of 6–10 rats sequentially sacrificed over a period of 24 h. As shown in Figure 3(a), 2h after bacterial challenge all the rats were infected and had similar vegetation bacterial titers irrespective of the infecting organism, indicating that all the lactococcal recombinants were comparably able to colonize the vegetations. However, the disease evolved differently later on. While the vegetation density of parent *L. lactis* pIL253 remained stable over time, the vegetation density of *L. lactis* vWbp recombinants increased whereas the vegetation density of Coa-positive lactococci decreased until eradication at 24 h. These results are in line with those obtained in rats infected with 10^7^ CFU and killed at 24 h post-challenge, where most of the vegetations were sterile (see Figure 2). Thus, Coa production by lactococci attached to the vegetations promoted active bacterial clearance from the fibrin-platelet clots.

To support the hypothesis of the platelet-mediated clearance of *L. lactis* Coa in the vegetations, freshly-prepared platelet-rich plasma (PRP) from rats was incubated with lactococcal cells and the number of viable cells was assessed after 4 and 24 h of incubation at 37°C. As shown in Figure 3(b), while viability of *L. lactis* piL253 and *L. lactis* vWbp did not change over time, numbers of *L. lactis* Coa were significantly reduced after 24 h incubation, supporting the notion that Coa triggers platelet-induced bacterial killing in the in the platelet-fibrin network.

### vWbp does not increase L. lactis IE in the presence of ClfA

**Figure 4.**
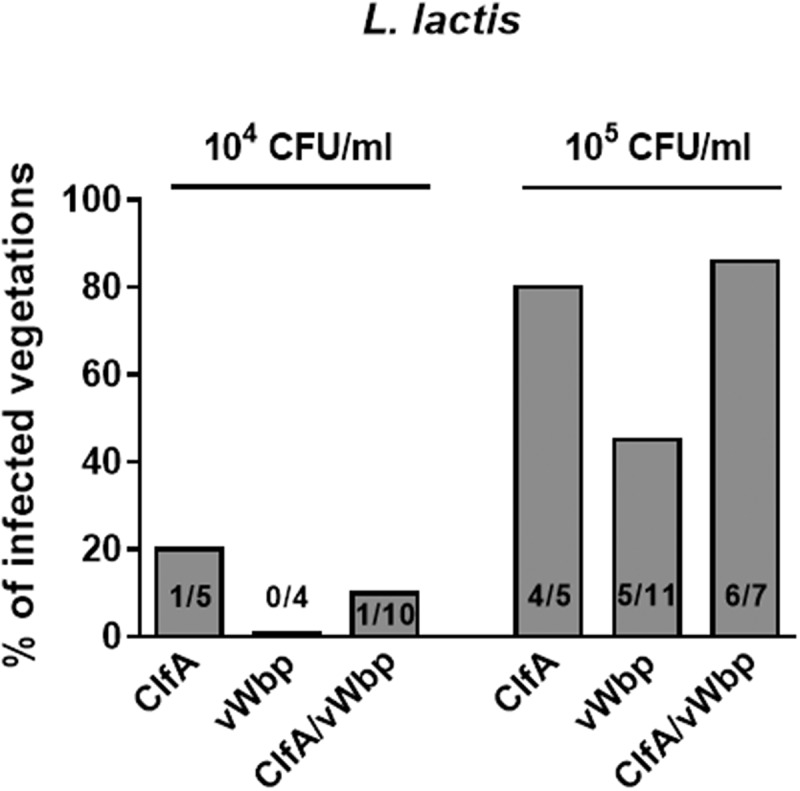
*vWbp* does not increase *L. lactis* IE in the presence of *clfA*. Experimental endocarditis induced by inoculation of parent *L. lactis* pIL253 and recombinant *L. lactis* expressing *clfA* or *vWbp* alone or in combination. Rats with catheter-induced aortic vegetations were challenged with 10^4^ or 10^5^ CFU of the indicated strains. The columns express the percentage of infected vegetations 24 h after inoculation. The number of infected/total number of vegetations per group is indicated at the bottom of the columns. No significant differences (*P *> 0.05) were observed when infection rates and bacterial burden in vegetations were compared.

Previous studies using recombinant *L. lactis* expressing ClfA or ClfA-defective *S. aureus* have demonstrated that ClfA plays a pivotal role in the initiation of experimental IE [6,7]. To further investigate the role of vWbp in the presence of ClfA, we generated a double *vWbp/clfA* recombinant *L. lactis* by inserting *vWbp* in *L. lactis* ClfA [6] and assessed its ability to induce IE. As shown in Figure 4, when rats were challenged with inocula containing 10^4^ CFU, *L. lactis* ClfA exhibited a poor valve infection rate, but co-expression of *vWbp* did not increase *L. lactis* valve infection. At higher inoculum (10^5^ CFU), *L. lactis* expressing *clfA* and *L. lactis* expressing both *clfA* and *vWbp* were more prone to induce experimental IE than *L. lactis* expressing *vWbp* alone (*P *= 0.19 and *P *= 0.08, respectively; Chi-squared test). However, due to the high infectivity of *L. lactis* ClfA (> 80% of vegetations), it was impossible to investigate whether the concomitant production of vWBP had a cooperative effect on promoting IE initiation.

All animals that developed endocarditis when inoculated with 10^5^ CFU/ml had similar vegetation bacterial densities, *i.e*. 5.73 ± 0.97 log_10_ CFU/g (*L. lactis* pIL253), 6.00 ± 1.05 log_10_ CFU/g (*L. lactis* Coa) and 5.59 ± 0.46 log_10_ CFU/g (*L. lactis* vWbp); (*P *> 0.05; one-way ANOVA).

### Neither Coa nor vWbp are necessary for the establishment of S. aureus IE when ClfA is present

In the light of the results obtained using the *L. lactis* surrogate expression system, we further investigated the ability of Coa and vWbp to colonize valve vegetations in the *S. aureus* background, in the presence or absence of ClfA. To this end, *S. aureus* Newman and isogenic mutants devoid of *coa, vWbp* or/and *clfA*, individually or in combination, were used. The defects in blood clotting of these mutants have already been described [16].

**Figure 5.**
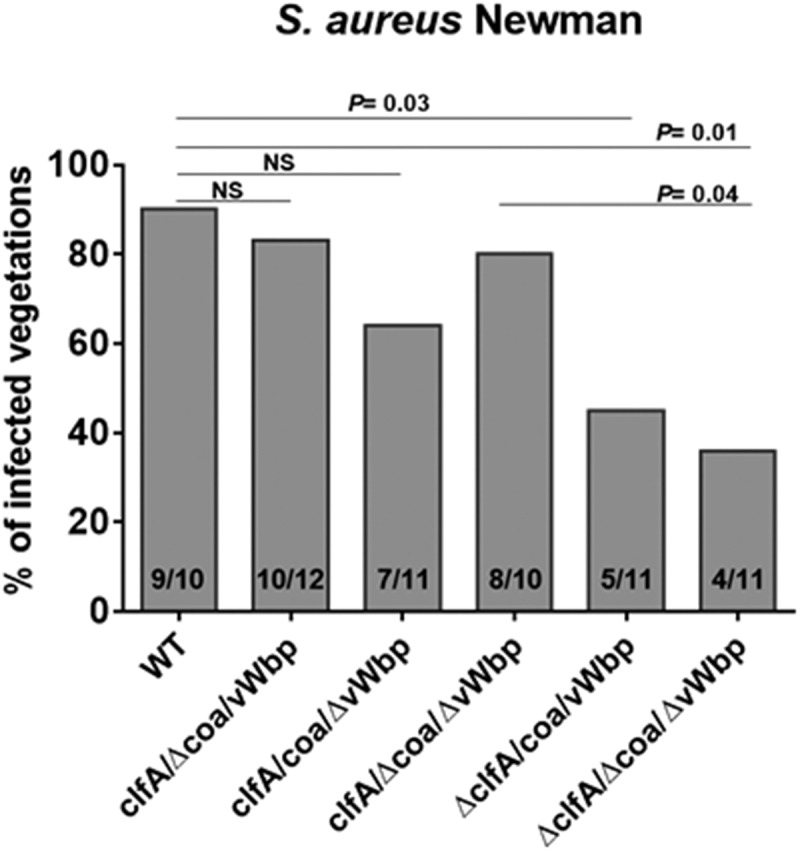
Neither *coa* nor *vWbp* significantly affects *S. aureus* Newman infectivity. Experimental endocarditis induced by inoculation of parent *S. aureus* Newman and isogenic mutant strains lacking *coa, vwbp* or *clfA* alone or in combination. Rats with catheter-induced aortic vegetations were challenged with 10^4^ CFU of the indicated *S. aureus* Newman strains. The columns express the percentage of infected vegetations 24 h after bacterial challenge. The number of infected/total number of vegetations per group is indicated at the bottom of the columns. Statistical comparisons were determined by the Chi-square test. NS, not significant.

Rats were challenged with different inocula of *S. aureus* Newman allowing quantification of infectivity [6]. Valve infection was evaluated 24 h after challenge as described above. As shown in Figure 5, deletion of either the *coa* or *vWbp* gene did not significantly decrease infectivity (83% and 64% infected vegetations, respectively). In contrast, the absence of *clfA* (*S. aureus* Δ*clfA*/*coa*/*vWbp*) led to a significant decrease in *S. aureus* infectivity (45% infected vegetations; *P *= 0.03 compared to parent), in line with the results reported in a previous work [6]. Moreover, inactivation of *coa* and *vWbp*, in addition to that of *clfA* (*S. aureus* Δ*clfA*/Δ*coa/*Δ*vWbp*), resulted in a slight but insignificant decrease of *S. aureus* infectivity (36% infected vegetations) as compared to that of the single *clfA*-defective mutant (45% infected vegetations). Of note, the bacterial densities in the vegetations of the animals that developed endocarditis were not significantly different, ranging from 7.24 to 8.89 log_10_ CFU/g. These results confirm that ClfA is the major determinant in the establishment *S. aureus* IE and suggest that its presence might outshine other virulence factors, such as vWbp in this particular animal model.

## Discussion

We here examined the role of the two *S. aureus* procoagulant factors (Coa and vWbp) in the initiation of IE. This study was based on the assumption that by triggering fibrin formation and platelet aggregation, two essential events in the vegetation development, Coa and vWbp might contribute to the establishment of IE. To test this hypothesis, we used the poorly pathogenic *L. lactis* as a surrogate vector and investigated the effects of heterologous expression of the *S. aureus* procoagulant factors on its ability to colonize heart valve vegetations, using a rat model of catheter-induced experimental endocarditis. We generated recombinant *L. lactis* strains and showed that they could express and efficiently secrete the *S. aureus* coagulases. Moreover, consistent with the work of McDevitt and others, who showed that Coa was partly retained on *S. aureus* surface [24], we observed that Coa, but not vWbp, is partially retained on *L. lactis* cell wall.

Unexpectedly, the expression of the *S. aureus* coagulases produced contrasting effects on the abilities of recombinant lactococci to infect damaged valves. On the one hand, we observed that Coa did not confer lactococci an increased ability to induce IE in rats, as assessed 24 h after challenge. These results are in agreement with previous studies showing that Coa is not involved in the initiation of *S. aureus* IE [6,20,21]. However, at that time the presence of vWbp was unknown and its presence might have compensated for and thus outshined the loss of Coa function. Remarkably, we also found that Coa not only failed to promote IE, but even made lactococci less infective. In fact, following the evolution of IE over 24 h we observed three profiles for the parent and the *vWbp* and *coa* recombinants. While vegetation densities of the parent remained stable, those of the *vWbp* recombinant increased whereas those of the *coa* recombinant decreased down to infection eradication. Yet, this effect was not due to reduced capability of Coa-positive lactococci to colonize the valves, as titers comparable to those of the parent lactococci and *L. lactis* vWbp were found in the vegetations 2 h after challenge.

Although a precise mechanism of *L. lactis* Coa clearing remains to be determined, one plausible hypothesis is that Coa could induce a progressive killing of lactococci by platelets present in the vegetations. Consistent to this notion, it was previously shown that platelets can release microbicidal proteins that kill bacteria within vegetations [25–27]. Moreover, it was also demonstrated that, during the early phase of infection, the induction of the coagulation cascade by bacteria leads to their entrapment and killing within the clot, likely by the generation of additional host antimicrobial peptides [28,29]. We observed that the ability of *L. lactis* Coa cells to coagulate plasma was much faster than that of *L. lactis* vWbp cells, requiring 100-times less bacteria to induce clot formation after 24 h (supplementary Figure S1). This, together with the fact that *L. lactis* Coa displayed a greater ability to aggregate platelets and to coagulate plasma than *L. lactis* vWbp supports the killing hypothesis of the *L. lactis* Coa strain in the platelet-fibrin vegetation meshwork.

The detrimental effects of Coa expression in the early steps of IE may explain the need for tight regulation in *S. aureus*. In this organism, coagulase is only transiently produced at the beginning of the exponential growth phase [30], a limited period of time that could be enough to promote fibrinogen-fibrin binding while avoiding overwhelming activation of platelets. This would be consistent with the observation by Panizzi et al [22], who showed that Coa production was restricted to the growing edge of mature vegetations at the interface with the blood stream in mice infected with *S. aureus* Newman, where bacteria are not yet clustered in stationary phase-like, or biofilm-like, conditions.

On the other hand, the present results show for the first time that the expression of vWbp in *L. lactis* might contribute to the establishment of IE. Recently, *S. aureus* and *Staphylococcus lugdunensis* were shown to bind directly to the blood glycoprotein von Willebrand factor (VWF) [31,32]. In case of *S. aureus*, binding to VWF is mediated by secreted vWbp, which in turn binds to *S. aureus* via cell-wall anchored ClfA [31,32]. Thus, VWF present in the vegetations may act as local ligand to trap circulating bacteria.

However, this scenario was unlikely in vWbp-producing lactococci, because recombinant vWbp was secreted and not attached to the bacterial surface. Therefore, the increased infectivity of these recombinants was more likely due to the vWbp procoagulant activity alone, via binding to VWF present in vegetations and entrapping the organisms within the valve lesions [33,34]. Moreover, the fact that vWbp lactococci were less pro-coagulant than their Coa-producing counter parts, plus the fact that vWbp was not attached to the bacterial wall, might explain a lower platelet proximity and a lower platelet-induced lethality.

In this study we also tested the role of ClfA in these constructs. ClfA binds to fibrinogen-fibrin and also promotes platelet activation. This is why it increases infectivity when produced in lactococci [4,7]. However ClfA also binds vWbp and bridges lactococci to VWF, as in *S. aureus* [31]. We asked whether this process would affect infectivity of either *cflA* or *vWbp* lactococcal recombinants, or *S. aureus* deleted in various *vWbp, coa* or *clfA* gene combinations. Using *S. aureus* Newman, a strain that does not express a functional FnbpA [35], we found that ClfA was the major determinant of infectivity, and that vWbp or Coa did not add any advantage in this particular model of IE. Thus, while vWbp had indeed some effect in promoting infectivity in lactococcal recombinants, it became dispensable in the presence of ClfA.

In sum, vWbp appears to play a marginal role in the initiation of *S. aureus* IE. However, while this assumption is true for the present IE experimental model, where pre-existing physical valve damage is the nidus of infection, it might be different in other scenarios, such as minimal endothelial lesions, which are associated with the presence of high VWF levels. In that case the role of vWbp, together with ClfA to bridge the bacterium to the inflamed endothelium, might become more prominent. Whether vWbp becomes the predominant player of *S. aureus* infection in such circumstances needs yet to be clarified *in vivo*.

## Materials and methods

### Bacterial strains and growth conditions

The bacterial strains used in this study are listed in Table 1. *L. lactis* were cultivated at 30°C in M17 broth (Becton Dickinson, Sparks, MD) or on M17 agar plates supplemented with 0.5% glucose. *S. aureus* were grown under static condition in tryptic soy broth or on tryptic soy agar (Becton Dickinson). *Escherichia coli* were grown at 37°C in Luria-Bertani medium (Becton Dickinson). When required, antibiotics were added to the media at the following concentrations: erythromycin 5 µg/ml for *L. lactis* and 500 µg/ml for *E. coli*, chloramphenicol 10 µg/ml for *L. lactis* and 30 µg/ml for *E. coli* and ampicillin 100 µg/ml for *E. coli*.

**Table 1.** Bacterial strains used in this study.

Strain *^a^*	Properties	Source or Reference
*L. lactis* subsp. *cremoris* 1363	Wild type	[4]
*L. lactis* pIL253	Parent strain carrying the empty vector pIL253	[4]
*L. lactis* Coa	Derivative of pIL253 expressing *S. aureus coa*	This study
*L. lactis* vWbp	Derivative of pIL253 expressing *S. aureus vWbp*	This study
*L. lactis* Coa/vWbp	Derivative of pIL253 expressing *S. aureus coa* and *vWbp*	This study
*L. lactis* ClfA	Derivative of pIL253 expressing *S. aureus clfA*	[4]
*L. lactis* ClfA/vWbp	Derivative of pIL253 expressing *S. aureus clfA* and *vWbp*	This study
*S. aureus* Newman	Wild type	[16]
*S. aureus* ∆*coa*	Derivative of Newman lacking *coa*	[6]
*S. aureus* ∆*vWbp*	Derivative of Newman lacking *vwbp*	[16]
*S. aureus* ∆*clfA*	Derivative of Newman lacking *clfA*	[16]
*S. aureus* ∆*coa*/∆*vWbp*	Derivative of Newman lacking *coa* and *vWbp*	[16]
*S. aureus* ∆*coa*/∆*vWbp/*∆*clfA*	Derivative of Newman lacking *coa, vWbp* and *clfA*	[16]
*E. coli* DH5α		Promega

*^a^* Coa: staphylocoagulase; vWbp: von Willebrand factor-binding protein;

ClfA: clumping factor A

### Plasmids and plasmid constructions

All the plasmids and primers used in this work are listed in supplementary Table S1. *S. aureus* genomic DNA was extracted as previously described [36]. *S. aureus* Newman *coa* and *vWbp* were PCR amplified using the forward primers *coa*-fw and *vWbp*-fw and the reverse primers *coa*-rv and *vWbp*-rv. The resulting PCR products were digested with SalI and PstI and ligated into the p*ori*23 vector, also digested with the same enzymes. The resulting constructs p*ori*23-*coa* and p*ori*23-*vWbp* containing *coa* and *vWbp* under the control of the lactococcal P23 constitutive promoter [37] were amplified in the *E. coli* DH5α intermediate host and purified using the Wizard® *Plus* SV Minipreps DNA Purification System (Promega, Madison, WI). The absence of mutations in the inserts was verified by commercial sequencing. The constructs were finally electroporated into *L. lactis* subsp. *cremoris* MG1363 cells using a protocol described previously [38].

### Characterization of the recombinant L. lactis strains

The functionality of recombinant *L. lactis* Coa and *L. lactis* vWbp was verified by assessing blood coagulation. Briefly, 100 μl containing 10^7^ CFU of *L. lactis* cells were added to 900 μl of citrated rat blood in polystyrene tubes. The tubes were incubated at 37°C for 24 h and blood coagulation was verified by tipping the tubes at a 45° angle [16]. For the clot inhibition tests, the direct thrombin inhibitor dabigatran (150 ng/ml, final concentration) [39] was added to citrated rat blood prior to addition of the bacteria.

### Quantification of platelet aggregation

The ability of *L. lactis* cells and cell-free supernatants to induce platelet aggregation *in vitro* by means of secreted and surface-associated coagulases was measured by light transmission aggregometry. Platelet-rich plasma (PRP) and platelet-poor plasma (PPP) were prepared as previously described [40]. Twenty microliters containing either *L. lactis* cells (10^7^ CFU) or cell-free supernatants of the same cultures were added to 180 μl of PRP in siliconized flat-bottom cuvettes. The light transmission of PRP without added bacteria and the light transmission of PPP were defined as 0% and 100% light transmission, respectively. Platelets were also tested with 10 μM ADP, used as positive control. Aggregation was recorded for 20 min. Three independent assays were performed.

### Titration of plasma clotting activity of L. lactis

To titrate the plasma clotting activity over time, 100 μl of different inoculum sizes (from 10^5^ to 10^8^ CFU) of *L. lactis* cells (serially diluted in PBS) were mixed with 100 μl of rat plasma in polystyrene tubes. After gently mixing, the tubes were incubated at 37°C and observed at different times (4, 8, 12, 24 and 36 h) until clot formation.

### Ethics statement

Animal experiments were carried out in accordance with the recommendations of the Swiss Federal Act on Animal Protection. All animal protocols were reviewed and approved by the Cantonal Committee on Animal Experiments of the State of Vaud (Permit Number: 879.9).

### Animal model of endocarditis

Catheter-induced sterile aortic vegetations were produced in female Wistar rats (180–200 g) as previously described [41]. Twenty-four hours after catheterization, animals were inoculated intravenously (i.v.) with increasing numbers of either *L. lactis* (ranging from 10^4^ to 10^8^ CFU/ml) or *S. aureus* (10^4^ CFU/ml). The catheter was left in place throughout the entire experiment. The cardiac vegetations were sterilely removed, weighed, homogenized and plated on M17 agar plates containing the appropriate antibiotic marker. Vegetation infection was evaluated 24 h later [6,7] and expressed as log_10_ CFU/g of vegetation. Aortic valve vegetations were considered positive when they contained > 2 log_10_ CFU/g, which corresponds to the lower limit of detection of growth. In certain experiments, designed to measure diseases progression, rats were sacrificed at different time points after i.v. challenge with high inoculum sizes (10^7^ CFU of *L. lactis*). Bacterial densities in the vegetations were measured as above.

### In vitro killing of L. lactis by platelets

PRP were prepared from anticoagulated rat blood as described above. *L. lactis* suspensions containing 10^5^–10^6^ CFU/ml were mixed with PRP at a 1:1 ratio in polypropylene tubes. To monitor killing activity of platelets, viable bacterial cells were enumerated after 4 and 24 h incubation at 37°C. In order to accurately count viable bacteria, samples were vortexed and mixed by pipetting up and down before plating to disrupt any visible aggregate. Experiments were performed in triplicate in two independent occasions.

### Statistical analysis

The incidences of valve infection were compared by the Fisher’s exact test or the Chi-squared test. Bacteria CFUs were compared by the one-way ANOVA followed by Tukey’s multiple comparisons test. A value of *P *< 0.05 was considered significant by using two-tailed significance levels. All statistical analyses were performed with the GraphPad Prism 6.0 program (www.graphpad.com).
