# Supplementary material for: Marginal role of von Willebrand factor-binding protein and coagulase in the initiation of endocarditis in rats with catheter-induced aortic vegetations
Source: Virulence. 2018 Oct 13;9(1):1615–24. doi: 10.1080/21505594.2018.1528845 (PMC7000203; doi:10.1080/21505594.2018.1528845)
Supplement: Supplemental Material [file kvir-09-01-1528845-g000.docx]

**Supplementary Table S1**. Plasmids and primers used in this study. Restriction sites are underlined.

| **Plasmids** |  | **Properties** |  | **Reference** |
| --- | --- | --- | --- | --- |
| p*ori23* |  | *erm*AM ori ColE1 P23 |  | [7] |
| p*ori23-coa* |  | *erm*AM ori ColE1 P23 *coA* |  | This study |
| p*ori23-vWbp* |  | *cat* ori ColE1 P23 *vWbp* |  | This study |
| p*ori23-clfA* |  | *erm*AM ori ColE1 P23 *clfA* |  | [7] |
|  |  |  |  |  |
| **Primers** |  |  |  |  |
| *coa*-fw |  | ACGCGTCGACGATTGGGCAATTACATTTTGG, SalI |  |  |
| *coa*-rv |  | AACTGCAGTCTTTGGATAGAGTTACAAACTTA, PstI |  |  |
| *vWbp*-fw |  | ATATGTCGACGTAAGGTAGGTTGTTAATTAGGG, SalI |  |  |
| *vWbp*-rv |  | ATATCTGCAGTGCAGCCATGCATTAATTATTTG, PstI |  |  |
